# Valorization of rice straw, sugarcane bagasse and sweet sorghum bagasse for the production of bioethanol and phenylacetylcarbinol

**DOI:** 10.1038/s41598-023-27451-4

**Published:** 2023-01-13

**Authors:** Rojarej Nunta, Charin Techapun, Sumeth Sommanee, Chatchadaporn Mahakuntha, Kritsadaporn Porninta, Winita Punyodom, Yuthana Phimolsiripol, Pornchai Rachtanapun, Wen Wang, Xinshu Zhuang, Wei Qi, Kittisak Jantanasakulwong, Alissara Reungsang, Anbarasu Kumar, Noppol Leksawasdi

**Affiliations:** 1 Cluster of Agro Bio-Circular-Green Industry (Agro BCG) & Bioprocess Research Cluster (BRC), School of Agro-Industry, Faculty of Agro-Industry, Chiang Mai University, Chiang Mai, 50100 Thailand; 2 Division of Food Innovation and Business, Faculty of Agricultural Technology, Lampang Rajabhat University, Lampang, 52100 Thailand; 3 Faculty of Agro-Industry, Chiang Mai University, Chiang Mai, 50100 Thailand; 4 Center of Excellence in Materials Science and Technology, Faculty of Science, Chiang Mai University, Chiang Mai, 50100 Thailand; 5 Guangdong Provincial Key Laboratory of New and Renewable Energy Research and Development, CAS Key Laboratory of Renewable Energy, Guangzhou Institute of Energy Conversion, Chinese Academy of Sciences, Guangzhou, 510640 People’s Republic of China; 6 Research Group for Development of Microbial Hydrogen Production Process, Khon Kaen University, Khon Kaen, 40002 Thailand; 7 Department of Biotechnology, Faculty of Technology, Khon Kaen University, Khon Kaen, 40002 Thailand; 8 Academy of Science, Royal Society of Thailand, Bangkok, 10300 Thailand; 9 Department of Biotechnology, Periyar Maniammai Institute of Science & Technology, Thanjavur, 613403 India

**Keywords:** Biochemistry, Biotechnology, Microbiology, Energy science and technology, Engineering

## Abstract

Open burning of agricultural residues causes numerous complications including particulate matter pollution in the air, soil degradation, global warming and many more. Since they possess bio-conversion potential, agro-industrial residues including sugarcane bagasse (SCB), rice straw (RS), corncob (CC) and sweet sorghum bagasse (SSB) were chosen for the study. Yeast strains, *Candida tropicalis*, *C. shehatae*, *Saccharomyces cerevisiae,* and *Kluyveromyces marxianus* var. *marxianus* were compared for their production potential of bioethanol and phenylacetylcarbinol (PAC), an intermediate in the manufacture of crucial pharmaceuticals, namely, ephedrine, and pseudoephedrine. Among the substrates and yeasts evaluated, RS cultivated with *C. tropicalis* produced significantly (p ≤ 0.05) higher ethanol concentration at 15.3 g L^−1^ after 24 h cultivation. The product per substrate yield (*Y*_eth/s_) was 0.38 g g^-1^ with the volumetric productivity (*Q*_p_) of 0.64 g L^−1^ h^−1^ and fermentation efficiency of 73.6% based on a theoretical yield of 0.51 g ethanol/g glucose. *C. tropicalis* grown in RS medium produced 0.303 U mL^−1^ pyruvate decarboxylase (PDC), a key enzyme that catalyzes the production of PAC, with a specific activity of 0.400 U mg^−1^ protein after 24 h cultivation. This present study also compared the whole cells biomass of *C. tropicalis* with its partially purified PDC preparation for PAC biotransformation. The whole cells *C. tropicalis* PDC at 1.29 U mL^−1^ produced an overall concentration of 62.3 mM PAC, which was 68.4% higher when compared to partially purified enzyme preparation. The results suggest that the valorization of lignocellulosic residues into bioethanol and PAC will not only aid in mitigating the environmental challenge posed by their surroundings but also has the potential to improve the bioeconomy.

## Introduction

As a consequence of the global population that is speculated to attain more than 9 billion by 2050 and 11 billion by 2100^1^, the adequate food supply is in question for the near future. To conquer this, scientific community has been exploiting various strategies on prevention of food spoilage^2,3^ and extending their shelf-life^4,5^ while seeking to identify useful microbes for food industry^6^. Regarding Sustainable Development Goal 2 (Zero Hunger), there has been a remarkable advancement in poultry, livestock and crop production, which additionally contribute to the evolution of food and agricultural wastes^7^. Food wastes can be recycled into commercially viable products, for instance, they are utilized as raw materials for the manufacture of bio-plastics and bio-fuels in addition to the extraction of value added components^8^. Food waste is also employed in industrial processes for the production of biofuels or biopolymers^9,10^. On the other hand, agro-industrial residues can be used for biomass to energy, mushroom production, cardboard/paper production, and other off-farm applications^11^. Although, they can be recycled for the manufacture of valuable items like chipboard, particleboard, bio-composites^12^ or other construction materials^13^, the volume of residues that these alternatives can currently use are a fraction of what is actually produced^11^. Thus, in many countries, in the absence of proper management and utilization practices for this huge quantity of residues, they are currently burnt or buried beneath the soil which leads to air and water pollution and global warming^14^. Open-air burning of residues contributes a fine particulate matter (PM) pollution, an important health risk factor that significantly contributes to mortality in several regions of the world including Southeast Asia. In 2019, a Global Burden of Disease (GBD) study classified PM2.5 exposure as the 6th global mortality risk factor^15^. The extent of agricultural waste burning and its catastrophic consequences on air quality is categorized to be the 7th leading mortality risk factor^15–17^ in Thailand, a major agricultural producer in Southeast Asia. This improper management has spawned an intense requirement to figure out strategies for timely utilization and valorization of agricultural residues for sustainability and food and health security^14^.

Since crop residues and agro-industrial wastes are lignocellulosic biomass composed of cellulose, lignin and hemicellulose, they can be used in the production of green energy and value-added products^18^ instead of burning. A biorefinery approach is a coherent and viable substitute to synthesize diverse bioproducts from lignocellulosic biomass. They are predominantly composed of cellulose, hemicellulose, and lignin^19^ which can be hydrolyzed either chemically or enzymatically to yield fermentable sugars, glucose and xylose, respectively, as well as *L-*arabinose^20,21^. In the concept of biorefinery, using anaerobic digestion, fermentation, and composting, we can convert the abundant waste biomass into biorefinery products such as biofuels, biofertilizers, bioplastics, enzymes, organic acids and other value-added chemicals^22–24^. Sugarcane bagasse, rice straw, corncobs and sweet sorghum bagasse which are the most common waste biomass generated by the agro-industries in large quantities annually are suitable materials for biorefinery conversion into value added products.

In the current scenario, the scientific community is working extensively to seek alternative, sustainable, and environment-friendly energy sources for combatting high energy demand. Bioethanol is one of the most suitable renewable, alternative energy sources to replace fossil fuels. Thus, researchers are active in producing bioethanol through microbial fermentation from inexpensive wastes such as lignocellulose^25,26^ for not only they are abundant but also do not compete with food production, thereby not affecting food security. In this regard, a biorefinery can be operated systematically to achieve zero waste production^27^ by manufacturing high-value-added chemicals in addition to bioethanol as it will also increase the profitability of bioprocess.

Pyruvate decarboxylase (PDC) is a key enzyme involved in bioethanol production by catalyzing pyruvate into acetaldehyde and carbon dioxide through a decarboxylation reaction^28^. PDC, in addition to decarboxylation, also carries out carboligation reaction wherein it bridges a carbon bond from benzaldehyde into the active acetaldehyde resulting in the production of *R–*phenylacetylcarbinol (PAC), a major precursor involved in the manufacture of pharmaceuticals, namely, ephedrine and pseudoephedrine. Ephedrine and pseudoephedrine are used to prevent or relieve wheezing associated with asthma^29^, to relieve low blood pressure during spinal or epidural anaesthesia^30^, in the management and treatment of clinically significant hypotension^31^, act as a decongestant which reduces nasal congestion^32^. From the literature, it is also clear that ephedrine is a pharmaceutical drug to exhibit several adrenaline actions and it is used in the preparation of obesity management and sport medicine^33^. Many researchers in recent times have also reported that the drug ephedrine could be a clinical neuroprotective candidate for treatment of cerebral ischemic stroke^34^ and brain injury^35^. Also, ephedrine could exhibit protective effects against chronic obstructive pulmonary disease^36^ and one of the standard choices of vasopressor agents to offset the consequences of life-threatening illnesses such as infection, heart failure, and anaphylaxis during surgical procedures^37,38^. Owing to its significant impact in medical field, the drug ephedrine is being listed under the WHO’s 21st edition of model list of essential medicines^39^. Such recent developments in medical field reveal that ephedrine has potential applications in the pharmaceutical sector and its global market is anticipated to rise at a considerable rate and will continue to play a significant role in the pharmaceutical industry and public and primary health care in the future. In this regard, being the precursor of ephedrine, the commercial production of PAC, which is valued at 146 USD/kg^40^, is done through decarboxylation of pyruvate followed by carboligation of benzaldehyde^41^ by transferring enzyme-bound “active acetaldehyde” onto benzaldehyde via a nucleophilic addition^42^.

Different species of yeasts, fungi and bacteria have been utilized for the above biotransformation process by several researchers^43–46^. In a study conducted by Rosche et al.^47^, 105 yeast strains were screened for PAC production and three species of *Candida* sp. were identified as the most interesting candidates as they produced higher PAC with low inactivation by benzaldehyde and acetaldehyde. The same authors in another study reported *Rhizopus javanicus* as a potential producer of PAC among 14 ethanol producing fungi based on higher yield and rapid growth^48^. Similarly, in our previous research, 50 microbial strains were screened for the production of ethanol and PAC and the results indicated that *C. tropicalis* was shown to be superior among other strains^49^. In another study reported by Miguez et al.^45^, *Kluyveromyces marxianus*, *Saccharomyces cerevisiae* and *S. pastorianus* were selected as best producers for PAC production. As far as the production of ethanol and PAC in an integrated process is concerned, the selection of appropriate microbial strains for PAC production not only depends on higher yield and tolerance against benzaldehyde toxicity, but also on its ability to utilize carbon sources available in the agro-industrial residues, able to produce appreciable amount of ethanol and biomass. Thus, in the present investigation, four yeast strains, *C. tropicalis* TISTR 5306, *C. shehatae* TISTR 5843, *S. cerevisiae* TISTR 5606 and *K. marxianus* var. *marxianus* TISTR 5057 were considered for the detailed study. The rationale for selecting *C. tropicalis* TISTR 5306 and *S. cerevisiae* TISTR 5606 was these strains performed well in producing ethanol and PAC in our previous studies^50,51^. *C. shehatae* TISTR 5843 and *K. marxianus* var. *marxianus* TISTR 5057 were included in this study for their abilities to consume pentose sugars.

Although several research data have been reported earlier on the production of ethanol from agro-industrial residues, this is the first study of its kind to evaluate different agro-industrial residues on simultaneous production of bioethanol and PDC enzyme. The study also investigated the influence of whole cells biomass and partially purified PDC enzyme on PAC production in a double-layer biotransformation system. This biorefinery approach will utilize the sugars present in the raw materials as carbon source for ethanol and PDC containing biomass generated in the fermentation could be used for the synthesis of PAC. Integrating the production of PAC and bioethanol will improve the overall profitability and productivity of both products. Therefore, in the present study, experiments were designed to select suitable raw material and yeast strain for optimal ethanol and PDC production as well as biotransformation studies for the assessment of PAC synthesis.

## Materials and methods

### Materials

Agricultural and agro-industrial waste materials including corncobs (CC) and rice straw (RS) obtained from Chiang Mai Provincial Livestock Office, sugarcane bagasse (SCB) from Kaset Thai International Sugar Corporation, and sweet sorghum bagasse (SSB) from a local farm in Saraphi District were washed with running tap water twice and sun-dried for 24 h. All agricultural materials except CC were chopped into small segments (about 1–3 cm length) while CC was attrited into small grits (about 0.3–0.5 cm diameter) with storage in dry condition at 25 °C. Cellulase from *Trichoderma reesei* was purchased from Vland Biotech Group Co. Ltd, China. The cellulase activity was determined by the method described by Ghose^52^ where the initial volumetric enzyme activity was 103 ± 3 FPU mL^−1^ prior to the study. *R*-PAC (based on Fischer Projection—an absolute configuration, which is equivalent to *L*-PAC) standard was procured from Toronto Research Chemicals, Toronto, Canada. All other standard chemicals and sugars were purchased from Sigma-Aldrich/Merck, Burlington, MA, USA. Reagents and solvents used in this study were analytical grades.

### Microorganisms

Ethanol producing yeasts procured from Thailand Institute of Scientific and Technological Research (TISTR), Bangkok, Thailand, were namely, *C. tropicalis* TISTR 5306, *C. shehatae* TISTR 5843, *S. cerevisiae* TISTR 5606 and *K. marxianus* var. *marxianus* TISTR 5057. The stock was maintained in 80% (v/v) glycerol at – 20 °C^53^. Yeasts were cultured on Yeast extract Peptone Dextrose (YPD) agar (yeast extract 10 g L^−1^, peptone 10 g L^−1^, agar 20 g L^−1^ and glucose 20 g L^−1^) and subsequently grown in YPD broth at 30 °C, 250 rpm for 24 h and assessed for cells viability. Yeast cells count was performed with a haemocytometer (Count Chamber, Model: PM MFR 650030, Haryana, India) by staining with 0.1% (w/v) methylene blue to distinguish live and death cells as described by Borzani and Vario^54^. All yeast strains had viable cells count of > 95% and were used as starter culture with 10% (v/v) inoculation^55^.

### Pretreatment and enzymatic saccharification

Raw materials were pretreated by suspending at 6.20% (w/v) in either distilled water or calcium hydroxide solution (1.84% w/v) and incubated at 100 °C for 4 h. The selection of calcium hydroxide for pretreatment was based on the results of our previous research that compared pretreatment strategies between calcium hydroxide, sulfuric acid, sodium hydroxide, and hydrogen peroxide with the optimal results in terms of overall sugars concentration, yield, and production cost (unpublished data). In addition, the advantages of calcium hydroxide pretreatment have been pointed out and successfully applied to other study from our group^56^. The pretreatment with distilled water was taken as control for comparison. The pretreated materials recovered by filtration was washed in water and suspended with sodium acetate buffer (50 mM, pH 4.8) at a 1: 5 (solid: liquid) ratio. A preliminary experiment consisting of different solid to liquid ratios were compared for the yield of sugars during pretreatment method and the solid to liquid ratio of 1:5 was found to be ideal resulting in higher sugars being released (unpublished data). Enzymatic hydrolysis of pretreated materials was performed by the addition of cellulase enzyme (10% v/v) at 50 °C for 48 h under shaking condition at 200 rpm as described by Wattanapanom et al.^56^. The hydrolysate was filtered using a double layered muslin cloth to remove coarse insoluble matter. The possible interference of insoluble fine particles matter that escaped muslin cloth filtration process at the beginning of microbial cultivation on determination of dried biomass concentration at specific time course could be removed by offset calculation of determined values relative to that at time zeroth. The type and concentration of sugars in the hydrolysate after enzymatic hydrolysis were analyzed by HPLC. The top three substrates with highest total sugars yield were chosen for the next experiment.

### Selection of suitable substrate for ethanol and yeast biomass production

As CC was shown to have a relatively lower level of total sugars from previous section of pretreatment and enzymatic saccharification, it was eliminated during selection process. The experiment was set up to evaluate the influence of substrates (SCB, RS and SSB) on microbial growth and ethanol production utilizing *C. tropicalis*. The cultivation conditions were similar to those described previously by Nunta et al.^51^. Briefly, a 10 mL seed inoculum from glycerol stock was grown in 250 mL Erlenmeyer flask with 90 mL hydrolysate supplemented with ammonium sulphate (8.52 g L^−1^) as a nitrogen source, at 250 rpm, 30 °C and sampling intervals at 0, 24, and 48 h in triplicate. The score ranking methodology was adapted from Nunta et al.^51^ and Tangtua et al.^49^ to evaluate the most suitable substrate for further experiments. The highest value of raw data considering all replicates for each criterion such as initial total sugars, ethanol and dried biomass concentration was assigned to a score of 100 and the other subsequent values were then converted proportionately to the same scale.

### Selection of suitable yeast for ethanol, biomass and PDC activity

In this experiment, the yeasts, *C. tropicalis*, *C. shehatae*, *S. cerevisiae* and *K. marxianus* were evaluated for the production of biomass, ethanol and PDC activity on the selected substrate from above section. Seed inoculum was prepared by inoculating yeasts into 50 mL of yeast culture medium in 250 mL Erlenmeyer flasks and incubated at 250 rpm for 24 h at 30 ± 1 °C. The cultivation medium of 450 mL of hydrolysate with added ammonium sulphate (8.52 g L^−1^) as nitrogen source was transferred with seed inoculum at 10% (v/v) in 1 L Erlenmeyer flask and incubated at 250 rpm for 48 h at 30 ± 1 °C^49^. As observed in our previous research^51^, this cultivation conditions provide initial aerobic environment sufficient to produce enough cells biomass and to initiate partial aerobic condition for subsequent ethanol production and PDC activity. The experiments were done in triplicate and the samples were drawn at 0, 24, and 48 h to evaluate sugars consumption as well as production of ethanol, dried biomass, and PDC activity. The related kinetic parameters were also determined during two intervals of 0–24 and 24–48 h. The score ranking strategy was used to assess the suitable yeast with selected substrate for subsequent biotransformation studies.

### Biotransformation process for the production of PAC in a two-layer liquid system

Biotransformation studies were carried out as described by Leksawasdi et al.^57^ and Gunawan et al.^58^ with slight modifications. The pre-frozen wet biomass of *C. tropicalis* at a whole cells concentration of 12.24 g L^−1^ was prepared with initial volumetric PDC activity of 1.29 ± 0.12 U mL^−1^. The partially purified PDC (0.32 ± 0.04 U mL^−1^) was also prepared and extracted from 12.24 g L^−1^ wet biomass based on previously published strategy^55^. The wet biomass or partially purified PDC as biocatalysts were added to the biphasic system comprised of an aqueous phase consisting of 125 mL 1 M phosphate buffer (pH 6.4/1 M H_3_PO_4_), pyruvate (240 mM), thiamine pyrophosphate (1 mM) and magnesium heptahydrate (1 mM) and an organic phase consisting of 125 mL of vegetable oil with benzaldehyde (200 mM)^59^. Biotransformation was initiated by stirring the mixture to form an emulsion of oil droplets in the aqueous phase at 10 °C for 6 h. The biotransformation process was carried out in aerobic condition, but it should be noted that the oxygen was not involved in PAC production process which relied upon decarboxylation and carboligation at active site of PDC. In addition, the biocatalysts used in current study were not live whole cells in growing or cultivation stage. Samples from oil and aqueous phases were withdrawn at 0, 5, 30, 60, 120, 180, 240, 300 and 360 min in quintuplicate and trichloroacetic acid (10% (w/v)) was added before centrifugation at 2822 × *g* for 5 min to separate the phases for further analyses.

### Analytical methods

For dried biomass estimation, the dried weight of the insoluble matter in the hydrolysate before inoculation was first measured by drying in a hot air oven (Daihan Lab Co., Ltd., Gangwon, Korea) at 105 °C until a constant weight was obtained. After inoculation, samples collected at intervals (0, 24 and 48 h) were centrifuged (Nuve, Model No. NF 200, Ankara, Turkey) at 2822 × *g* for 15 min to separate supernatant and pellet. The pellet was then washed with distilled water and analyzed for total dried weight which includes insoluble matter and biomass weight. The initial insoluble matter dried weight was subtracted from this total dried weight value to determine actual dried biomass weight^60^. The supernatant was analyzed for pH, total soluble solids (TSS), sugars (cellobiose, glucose, xylose, arabinose), ethanol, and acetic acid concentration. The pH was measured with a digital pH meter (Eutech Instruments, Model pH 510, Nijkirk, Japan) and the TSS (°Brix) was determined by a hand-held refractometer (Atago, Model No. N-1α, Tokyo, Japan). Sugars, ethanol and acetic acid concentration were analyzed using HPLC (Agilent Technologies, Santa Clara, California) as described by Khemacheewakul et al.^61^ with the following conditions: 5 mM sulphuric acid in distilled water as mobile phase with Aminex ® HPX-87H Ion Exclusive column and refractive index detector (RID) at 0.75 mL min^−1^ flow rate. The temperature of the column oven was set at 40 °C. Kinetic parameters like volumetric productivity of ethanol (*Q*_p_), specific total sugars consumption rate (q_s,tot_), specific growth rate (µ), yield of ethanol produced over total sugars consumed (*Y*_eth/s_), yield of acetic acid produced over total sugars consumed (*Y*_ace/s_), and yield of dried biomass produced over total sugars consumed (*Y*_x/s_) were calculated based on previously published work^51,53^. Similarly, the fermentation efficiency (FE%) was calculated using the established formula based on glucose^62^ and shown in supplementary section.

For preparation of partial purified PDC, a portion of yeast wet biomass (12.24 g) recovered from the above cultivation at 48 h at 1 L basis was used as described by Tangtua et al.^63^. Briefly, the biomass was added to citrate buffer (200 mM, pH 6.0) with glass beads (425–600 μm) at a 1: 1 ratio (w/w) to yeast cells and vortexed for 1 min to rupture the cells with intermittent cooling on ice for 1 min. Vortexing was repeated thrice and the resultant slurry was centrifuged at 2822 × *g* at 4 °C for 15 min to remove the pellet. The supernatant obtained was added with precooled (− 20 °C) acetone at 30% and left at 4 °C for overnight to precipitate the enzyme. Later, the acetone fraction was centrifuged at 12,122 × *g* at 4 °C for 15 min to collect the precipitate and the supernatant was added with acetone at 40% (v/v) and left at 4 °C overnight. The precipitation-centrifugation cycle was repeated with 50 and 60% acetone and the precipitates were pooled together and redissolved in citrate buffer (200 mM), stored at 4 °C for 4 h to evaporate any residual acetone. The resultant solution was assessed for PDC enzyme activity^51^ and protein concentration^64^ and utilized for the biotransformation process. PDC activity was measured as a formation of PAC in 20 min at 25 °C from 80 mM benzaldehyde and 200 mM pyruvate in carboligase buffer^43^. One-unit carboligase activity was defined as the amount of enzyme that could produce 1 µmol PAC from pyruvate and benzaldehyde per min at pH 6.4 and 25 °C as specified by Rosche et al.^43^. The protein concentration was determined according to Bradford assay, with bovine serum albumin as a protein standard^65^ and specific carboligase activity was expressed as a unit of enzyme per milligram protein (U mg^−1^). PAC, benzaldehyde, benzoic acid and pyruvate were determined by HPLC^49,50^ (Agilent Technologies) at 283 nm using 32% (v/v) of acetonitrile and 0.5% (v/v) of acetic acid in distilled water as mobile phase and Altima™ C8 column with diode array detector (DAD). The flow rate was set at 1.0 mL min^−1^ and the temperature of the detector was set at 28 °C.

### Statistical analysis

The results were expressed as mean ± standard error and analyses were performed with Statistical Packages for the Social Sciences (SPSS, version 17.0) using one-way analyses of variance (ANOVA). Statistical differences among means were determined using Duncan’s multiple comparisons and considered significant at p ≤ 0.05^60,61^.

## Results and discussion

### Selection of substrate for bioethanol production

The raw materials such as CC, SCB, SSB and RS used in the present study were the major agricultural and agro-industrial wastes in Thailand and classified as important lignocellulosic materials. The initial pretreatment was required to release the sugars from the raw materials to be used as a carbon source by yeasts^66^.

It was found that the raw materials upon pretreatment released varying concentrations of sugars as shown in Table 1. Pretreatment with 1.84% (w/v) calcium hydroxide followed by enzymatic digestion yielded significantly (p ≤ 0.05) higher sugars concentration compared to water treatment with enzymatic digestion. RS was found to release the highest sugars concentration with 68.9 ± 0.63 g L^−1^ followed by SSB (51.8 ± 0.45 g L^−1^), SCB (48.4 ± 0.17 g L^−1^) and CC (38.9 ± 0.44 g L^−1^). It was found that pretreatment with calcium hydroxide followed by enzymatic treatment released 23% (w/w) sugars from RS which was 44—64% higher when compared to other substrates subjected for similar treatment. Calcium hydroxide pretreatment followed by the enzymatic treatment of CC released the least quantity of sugars into hydrolysate when compared to any other substrate. In contrast to the present results, Sumphanwanich et al.^67^ compared CC, SCB and RS in the release of sugars upon dilute acid treatment, and found that CC yielded higher sugars than other lignocellulosic materials compared. The high hemicellulose and lignin contents of CC when compared to other materials might have hindered the action of cellulase during enzymatic treatment, thus decreased the efficiency of hydrolysis to release fermentable sugars. Therefore, CC was excluded from further experiments.

**Table 1:** Sugar content of raw materials after chemical and enzymatic pretreatment.

Substrate	Pre-treatment	Sugar concentration (g L^−1^)					Total sugars released (g g^−1^ of raw material)
		Cellobiose	Glucose	Xylose	Arabinose	Total sugars	
SCB	H_2_O + Cellulase	1.02^G^ ± 0.01	10.0^F^ ± 0.49	5.11^D^ ± 0.20	1.52^E^ ± 0.02	17.7^F^ ± 0.70	0.077^EF^ ± 0.003
	Ca(OH)_2_ + Cellulase	**8.76**^**A**^** ± 0.05**	28.0^C^ ± 0.09	10.9^C^ ± 0.17	0.795^F^ ± 0.026	48.4^C^ ± 0.17	0.137^D^ ± < 0.001
SSB	H_2_O + Cellulase	2.45^E^ ± 0.02	13.2^E^ ± 0.04	2.54^E^ ± < 0.01	2.07^C^ ± < 0.001	20.3^E^ ± 0.03	0.069^F^ ± < 0.001
	Ca(OH)_2_ + Cellulase	5.12^C^ ± 0.04	30.8^B^ ± 0.29	13.2^B^ ± 0.18	2.71^B^ ± 0.02	51.8^B^ ± 0.45	0.155^C^ ± 0.001
RS	H_2_O + Cellulase	1.23^F^ ± 0.19	16.0^D^ ± 1.16	2.78^E^ ± 0.15	1.85^D^ ± 0.02	21.9^E^ ± 1.44	0.083^E^ ± 0.005
	Ca(OH)_2_ + Cellulase	7.50^B^ ± 0.18	**44.5**^**A**^** ± 0.37**	13.3^B^ ± 0.12	**3.60**^**A**^** ± 0.03**	**68.9**^**A**^** ± 0.63**	**0.231**^**A**^ ± **0.002**
CC	H_2_O + Cellulase	0.35^H^ ± 0.05	0.00	0.00	0.00	0.35^G^ ± 0.05	0.002^D^ ± < 0.001
	Ca(OH)_2_ + Cellulase	3.59^D^ ± 0.03	18.4^D^ ± 0.29	**14.9**^**A**^** ± 0.12**	2.00^C^ ± 0.02	38.9^D^ ± 0.44	0.164^B^ ± 0.002

Numbers with the same uppercase alphabet indicated no significant difference (p > 0.05) for comparison of the same column. Bold data indicated the best average value(s) within each column.

SCB, sugarcane bagasse; SSB, sweet sorghum bagasse; RS, rice straw; CC, corncob.

**Figure 1:**
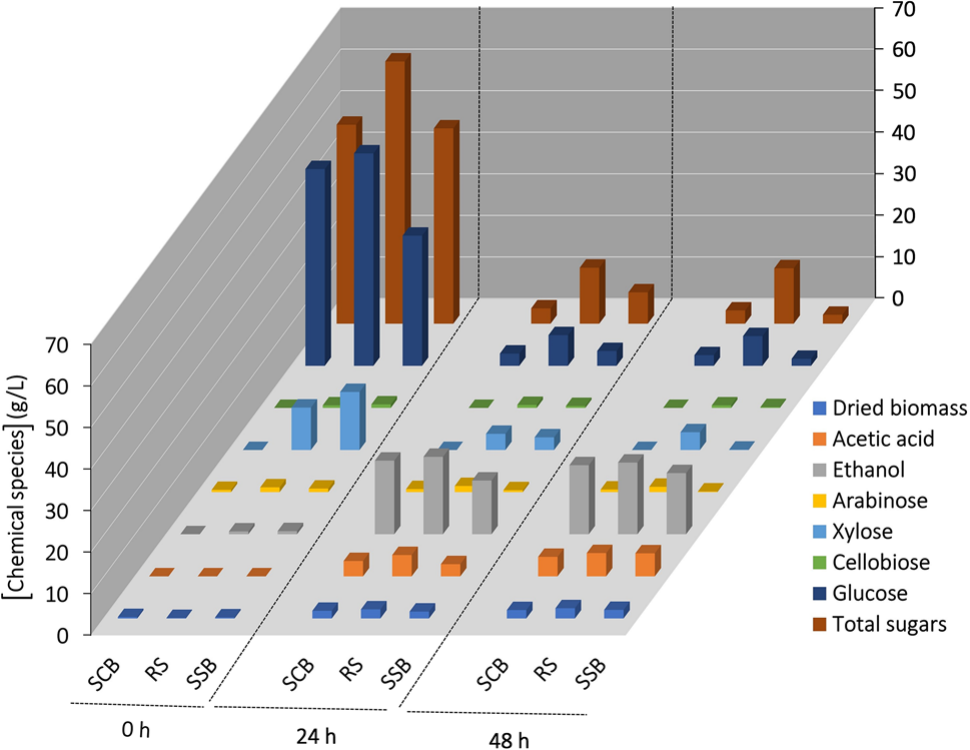
Kinetic profiles of total sugars, individual sugars (cellobiose, glucose, xylose and arabinose), ethanol, acetic acid and dried biomass concentration levels during cultivation of *C. tropicalis* TISTR 5306 on different substrates, sugarcane bagasse (SCB); rice straw (RS); sweet sorghum bagasse (SSB). The standard error in all cases were either within the sensitivity limit of detection procedures or less than 10%.

Further, *C. tropicalis*, a yeast capable of producing ethanol and PAC was utilized to screen suitable substrate among SCB, RS and SSB. A submerged cultivation was carried out and samples were analyzed at 0, 24, and 48 h intervals. In the present study, *C. tropicalis* grown in RS resulted in a significant (p ≤ 0.05) increase of 22% and 19% in dried biomass yields at the end of 48 h when compared to SCB or SSB used as substrates respectively (Fig. 1 and Supplementary Table S1). In addition, a significant (p ≤ 0.05) drop in pH was observed in all substrates at the end of 48 h when compared to their initial counterparts. Similarly, a significant (p ≤ 0.05) reduction of 40%, 49%, and 52% in total soluble solids were also observed at the end of 48 h for SCB, RS and SSB, respectively, when compared to their initial counterparts (Supplementary Table S1). This reduction in soluble solids is correlated with the utilization of carbon sources to synthesize energy while nitrogen was used in the syntheses of enzymes, proteins, deoxyribonucleic acid (DNA), and ribonucleic acid (RNA)^68^.

As shown in Fig. 1, the initial total sugar concentration was 47.9 ± 0.3, 63.1 ± 3.1 and 47.0 ± 1.0 g L^−1^ and during cultivation the sugars consumed were by 92%, 79% and 84% after 24 h and 94%, 79%, and 95% at the end of 48 h, respectively, for SCB, RS and SSB. The maximum ethanol production occurred at 24 h for SCB and RS whereas it was 48 h for SSB with 17.7 ± 0.2, 18.6 ± 0.2, and 14.7 ± 0.5 g L^−1^ respectively. Other kinetic parameters such as *Q*_p_, *µ*, *q*_s,tot_, *Y*_eth/s_, *Y*_ace/s_, and *Y*_x/s_ were presented in Supplementary Table S2. The *Q*_p_ was found to be 0.74, 0.78 and 0.31 g L^−1^ h^−1^ for SCB, RS and SSB respectively. From the results, it can be noted that the *Y*_eth/s_ during cultivation of *C. tropicalis* in different substrates were between 0.33 and 0.40 g ethanol produced g^−1^ sugars consumed for the initial 0–24 h of cultivation and 0.33 and 0.37 during 0–48 h. The highest *Y*_eth/s_ was obtained with SCB and RS with no significant difference (p > 0.05) in the yield.

From the score ranking analysis (Table 2), RS was found to be superior in terms of ethanol and biomass production and selected for further studies. In contrast to the results, Sumphanwanich et al.^67^ stated that the ethanol production by *S. cerevisiae* on RS hydrolysate was lower when compared to bagasse and CC. However, their results were corresponding to the initial sugar content of substrates. In the present study, RS was found to have the highest initial sugar content compared to other substrates to yield higher ethanol.

**Table 2:** Score ranking analysis to select suitable substrate for cultivation.

Substrate	(A)	(B)	(C)	(D)	Score for (A) (100 points)	Score for (B) (100 points)	Score for (C) (100 points)	Score for (D) (100 points)	Total Score (400 points)
	Total sugars after enzymatic treatment (g L^−1^)	Total sugars released (g g^−1^ raw material)	Ethanol concentration (g L^−1^)	Dried biomass concentration (g L^−1^)					
SCB	48.4^C^ ± 0.17	0.137^C^ ± < 0.001	16.6^A^ ± 0.26	2.02^B^ ± 0.06	69.3^C^ ± 0.2	58.6^C^ ± 0.2	93.7^A^ ± 1.5	82.0^B^ ± 2.5	304^B^ ± 2
RS	**68.9**^**A**^** ± 0.63**	**0.231**^**A**^ ± **0.002**	**17.2**^**A**^** ± 0.24**	**2.46**^**A**^** ± 0.01**	**98.7**^**A**^** ± 0.9**	**98.7**^**A**^** ± 0.9**	**97.3**^**A**^** ± 1.4**	**99.5**^**A**^** ± 0.5**	**394**^**A**^** ± < 1**
SSB	51.8^B^ ± 0.45	0.155^B^ ± 0.001	14.7^B^ ± 0.50	2.06^B^ ± 0.06	74.3^B^ ± 0.6	66.1^B^ ± 0.6	83.1^B^ ± 2.8	83.3^B^ ± 2.4	307^B^ ± 2

Numbers with the same uppercase alphabet indicated no significant difference (p > 0.05) for comparison of the same column. Bold data indicated the best average value(s) within each column. Score ranking was calculated taking account of all the replicates of each criterion in Microsoft Excel spreadsheet.

SCB, sugarcane bagasse; RS, rice straw; SSB, sweet sorghum bagasse.

### Selection of yeast for bioethanol and PDC production

**Figure 2:**
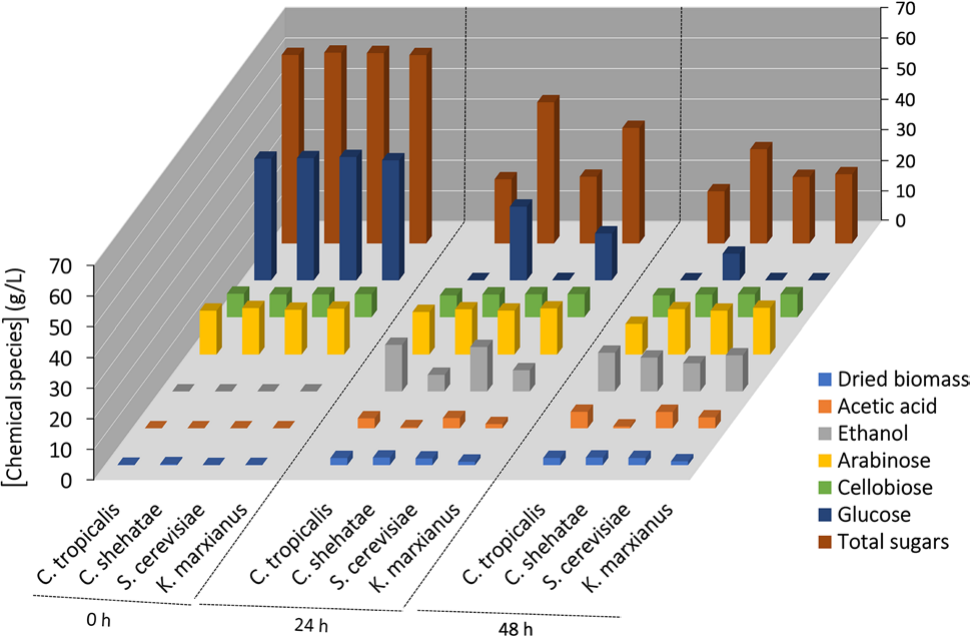
Kinetic profiles of total sugars, individual sugars (cellobiose, glucose and xylose), ethanol, acetic acid and dried biomass concentration levels during cultivation of different yeasts namely *C. tropicalis* TISTR 5306, *C. shehatae* TISTR 5843, *S. cerevisiae* TISTR 5606 and *K. marxianus* var. *marxianus* TISTR 5057 with rice straw as substrate. The standard errors in all cases were either within the sensitivity limit of detection procedures or less than 10%.

The yeasts, *C. tropicalis*, *C. shehatae*, *S. cerevisiae* and *K. marxianus*, were assessed in this study as there is a possibility that the PDC enzymes from different ethanol-producing yeasts will have different enzyme activity. *S. cerevisiae* is the most popular and superior yeast strain used to produce ethanol as it can withstand high ethanol concentrations compared to many other yeasts^69^. Despite this, the yeasts *C. tropicalis*, *C. shehatae* and *K. marxianus* were included in the study as they can utilize pentose sugars (xylose and arabinose), which *S. cerevisiae* cannot utilize, in addition to hexose sugars (sucrose and glucose). Moreover, the yeast *C. tropicalis* is resistant to lignin-derived degradative substances which have an inhibitory effect on yeast growth^70–73^. Thus, the above four yeasts were evaluated for their potential by fermenting RS substrate and the changes in parameters that occurred during cultivation are presented in Fig. 2 and Supplementary Table S3. The data for pH and TSS is presented with Supplementary Table S3. There was a significant (p ≤ 0.05) decrease observed in pH during cultivation from 0 to 24 h and further a slight decrease between 24 and 48 h. The initial soluble solid content was between 12.5 ± 0.3 and 12.7 ± 0.07°Brix among the yeasts studied. *C. tropicalis* showed maximum utilization of solids after 48 h of cultivation when compared to other yeasts and this is evident from the analysis of total sugars consumption after 48 h. There was no significant (p > 0.05) difference observed in dried biomass concentration between *C. tropicalis*, *C. shehatae* and *S. cerevisiae* while a lower biomass production was seen with *K. marxianus* compared to other yeasts after 48 h of cultivation. While other yeasts produced *Y*_x/s_ in the range of 0.05 ± 0.01 to 0.08 ± 0.01 g g^−1^, *K. marxianus* produced only 0.03 ± 0.01 g biomass produced g^−1^ sugars consumed.

The initial total sugar concentration before cultivation was found to be 61.6 ± 0.6, 62.4 ± 1.4, 62.2 ± 0.3 and 61.6 ± 0.7 g L^−1^ for *C. tropicalis*, *C. shehatae*, *S. cerevisiae* and *K. marxianus* respectively. It was observed that *C. tropicalis* and *S. cerevisiae* consumed 66.0% and 65.0% of total sugars to produce maximum ethanol after 24 h of cultivation with the concentration levels of 15.2 ± 0.3 and 14.5 ± 0.08 g L^−1^ ethanol, respectively. However, *C. shehatae* and *K. marxianus* produced maximum ethanol concentration only after 48 h of cultivation with the consumption of 50.6% and 63.2% total sugars to 11.1 ± 0.27 and 11.8 ± 0.17 g L^−1^ ethanol, respectively.

The comparison of kinetics is presented in Supplementary Table S4. The *Q*_p_ was found to be 0.64, 0.23, 0.61 and 0.25 g L^−1^ h^−1^ for *C. tropicalis*, *C. shehatae*, *S. cerevisiae* and *K. marxianus* respectively. The *Y*_eth/s_ obtained with *C. tropicalis* were found to be 8.6, 5.5 and 31.0% higher than that of *C. shehatae*, *S. cerevisiae* and *K. marxianus,* respectively. after 24 h of cultivation. The FE from this experiment was calculated to be 74.5%, 70.6%, 68.6%, and 58.8%, respectively for *C. tropicalis*, *S. cerevisiae*, *C. shehatae*, and *K. marxianus* based on a theoretical yield of 0.511 g ethanol g^−1^ glucose^74^. The higher ethanol yield with *C. tropicalis* over other yeasts is attributed to the tolerability of former yeast against temperature, ethanol, and lignin-like toxic polyphenols, moreover the ability to utilize pentose sugars presented in hemicellulose of agro-industrial products^70^.

With *C. tropicalis* and *S. cerevisiae*, the concentration of ethanol declined further at 48 h of cultivation with the depletion of glucose in the medium. This might be due to when glucose becomes scarce, ethanol produced during cultivation is used as a carbon source, requiring a shift to respiration as an adaptation which results in massive reprogramming of gene expression^75^. This might have resulted in the formation of acetic acid after 24 h of cultivation. The acetic acid yield (*Y*_ace/s_) was although higher for *S. cerevisiae* at the end of cultivation, it was not significantly different among the yeasts except *C. shehatae* (Supplementary Table S4). *C. tropicalis* and *S. cerevisiae* when grown in glucose deficient medium might have converted acetaldehyde generated from ethanol to acetic acid by the action of aldehyde dehydrogenase^76^. In future, reducing byproducts formation especially the acetic acid is a special concern in developing a competitive bioprocess in deciding the overall thrift of the process^77^.

There was only a negligible reduction in xylose observed in *C. tropicalis* during 0–24 h cultivation indicating that pentose-fermenting yeasts especially *Candida* sp. preferentially utilize glucose in mixtures of pentose and hexose sugars because of severe repression of pentose sugars catabolism^78^. Once glucose has been consumed, enzymes for pentose sugars catabolism are synthesized as evident in the present study where 30% (w/v) xylose was utilized during 24–48 h (Fig. 2). However, the rest of the yeasts failed to consume xylose as they prefer other hexose sugars or pentose sugars rather than xylose for their growth^79^. Similarly, none of the yeast used in the study could utilize cellobiose efficiently as they lack both a cellobiose transporter and a β-glucosidase capable of hydrolyzing cellobiose into glucose^80^. However, Zheng et al.^81^ demonstrated that *C. molishiana* was able to utilize cellobiose in addition to glucose and produce ethanol. The accumulation of cellobiose in the present study might be due to the inhibitory action of glucose by a strong product feedback inhibition. For instance, both endoglucanase and ß-glucosidase activities are required to break down cellulose into cellobiose and cellobiose into glucose, respectively. However, accumulation of glucose can inhibit ß-glucosidase and cellobiose can inhibit endoglucanase activity^82^. Thus, usage of a synergic ratio of endoglucanase and β-glucosidase can yield a high amount of glucose and thereby ethanol. This will also overcome glucose scarcity in the medium and improve ethanol to acetic acid ratio.

**Figure 3:**
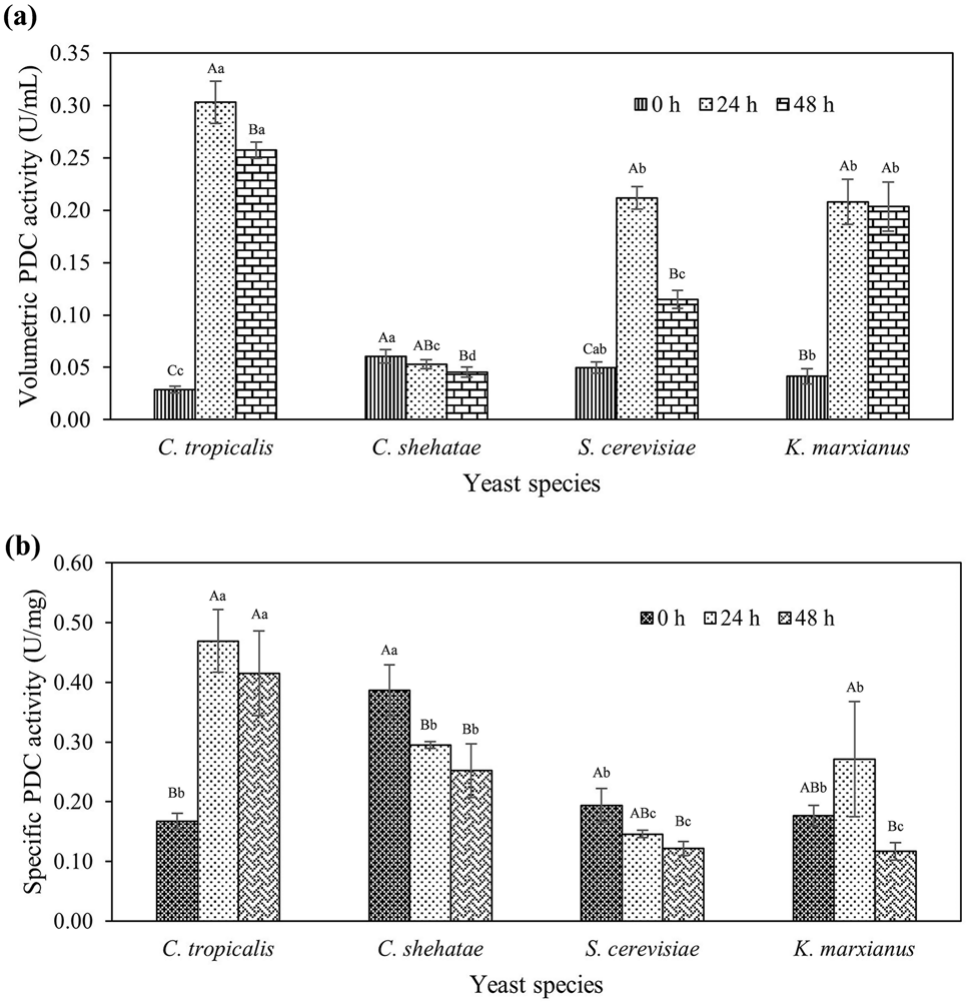
Intracellular pyruvate decarboxylase (PDC) activity of yeasts during cultivation with rice straw as substrate (**a**) Volumetric PDC enzyme activity of cells lysate; (**b**) Specific PDC enzyme activity of cells lysate. Error bars indicate the standard error from the mean (n = 3). Means with different uppercase alphabets indicated a significant difference (p ≤ 0.05) between time intervals of the same yeast species. Means with different lowercase alphabets indicated a significant difference (p ≤ 0.05) between respective time points among yeast species.

The yeasts recovered from the cultivation broth by centrifugation were lysed and analyzed for PDC activity. Figure 3 and Supplementary Table S5 show the enzyme and specific activity of PDC in the biomass of *C. tropicalis*, *C. shehatae*, *S. cerevisiae* and *K. marxianus*. The enzyme concentration was found to be higher during the initial stage of cultivation (up to 24 h) with a further decrease seen after 48 h cultivation. Among the yeasts, a higher concentration was observed in *C. tropicalis* with 0.303 ± 0.020 U mL^−1^ and specific activity of 0.469 ± 0.053 U mg^−1^ of protein after 24 h cultivation. In contrast, *C. shehatae* possessed the least volumetric enzyme activity with 0.053 ± 0.004 U mL^−1^ which was 6-folds lesser when compared to *C. tropicalis* after 24 h of cultivation.

From the score ranking analysis as shown in Table 3, *C. tropicalis* was found to be superior for ethanol, biomass production and PDC enzyme activity with a total score of 368 ± 7 out of 400 followed by *C. shehatae*, *K. marxianus* and *S. cerevisiae*. Thus, *C. tropicalis* was subjected to further biotransformation studies to synthesize PAC from the precursors, pyruvate and benzaldehyde.

**Table 3:** Score ranking analysis to select suitable yeast for cultivation.

Yeast	(A)	(B)	(C)	(D)	Score for (A) (100 points)	Score for (B) (100 points)	Score for (C) (100 points)	Score for (D) (100 points)	Total Score (400 points)
	Ethanol concentration (g L^-1^)	Dry biomass concentration (g L^-1^)	PDC enzyme activity value (U mL^−1^)	Specific activity values of the PDC enzyme (U mg^-1^ protein)					
*C. tropicalis*	**12.7**^**A**^** ± 0.23**	2.38^A^ ± 0.15	**0.257**^**A**^** ± 0.008**	**0.415**^**A**^** ± 0.041**	**97.4**^**A**^** ± 1.8**	88.8^A^ ± 5.6	**96.4**^**A**^** ± 2.9**	**85.4**^**A**^** ± 8.5**	**368**^**A**^** ± 7**
*C. shehatae*	11.1^C^ ± 0.27	**2.50**^**A**^** ± 0.12**	0.045^D^ ± 0.003	0.252^B^ ± 0.026	85.2^C^ ± 2.1	**93.3**^**A**^** ± 4.7**	17.0^D^ ± 1.0	51.9^B^ ± 5.4	247^B^ ± 12
*S. cerevisiae*	9.22^D^ ± 0.13	2.37^A^ ± 0.19	0.115^C^ ± 0.009	0.121^C^ ± 0.012	71.0^D^ ± 1.0	88.3^A^ ± 6.9	43.1^C^ ± 3.2	25.0^C^ ± 2.5	227^C^ ± 10
*K. marxianus*	11.8^B^ ± 0.17	1.29^B^ ± 0.02	0.203^B^ ± 0.024	0.117^C^ ± 0.008	91.0^B^ ± 1.3	48.0^B^ ± 0.7	76.3^B^ ± 8.8	24.0^C^ ± 1.7	239^BC^ ± 10

Numbers with the same capital alphabet indicated no significant difference (p > 0.05) for comparison of the same column. Bold data indicated the best average value(s) within each column. PDC: pyruvate decarboxylase. Score ranking was calculated taking account of all the replicates of each criterion in Microsoft Excel spreadsheet. For example: the highest value for raw data of column A (ethanol concentration) was 12.99 g L^−1^ among all the yeasts (the tabulated value of 12.7 g L^−1^ was an average value from three raw data which also included the highest value of 12.99 g L^−1^). All other values were then divided by maximum value of 12.99 and multiplied by 100 to get the respective score. Three replicate values of ethanol concentration for *S. cerevisiae* were 9.10, 9.08 and 9.47 g L^−1^. The score is calculated as (9.10/12.99*100) = 70.0, (9.08/12.99*100) = 69.9 and (9.47/12.99*100) = 72.9 for each replicate respectively. The scores of each replicate of the same yeast were added before calculating an average score. Therefore, for *S. cerevisiae* the average score (71.0) was calculated by (70.0 + 69.9 + 72.9)/3 = 71.0.

### Biotransformation process to produce PAC using whole cells biomass and partially purified enzyme as biocatalysts

Biotransformation studies were conducted in a two-layer liquid system with either whole cells biomass of yeast *C. tropicalis* or its partially purified enzyme. The reason to have biotransformation in a double-layer system is that the precursor, benzaldehyde, soluble only in the organic phase will prevent its interaction with PDC presents in the aqueous phase, thereby preserving the stability of the enzyme^83^. There are several solvents including octanol, heptanol, hexanol, butanol and many that can be used as organic phase system for the biotransformation process^50^. However, due to their higher costs, a vegetable oil was used as an organic phase in the present study to cut down the production cost. Although MOPS buffer used in the aqueous phase might help maintain the stability of the PDC enzyme, its relatively high cost will hinder its application in an industrial scale-up. Thus, the phosphate buffer used in the present study could be a suitable low-cost replacement for the MOPS buffer^61^.

**Figure 4:**
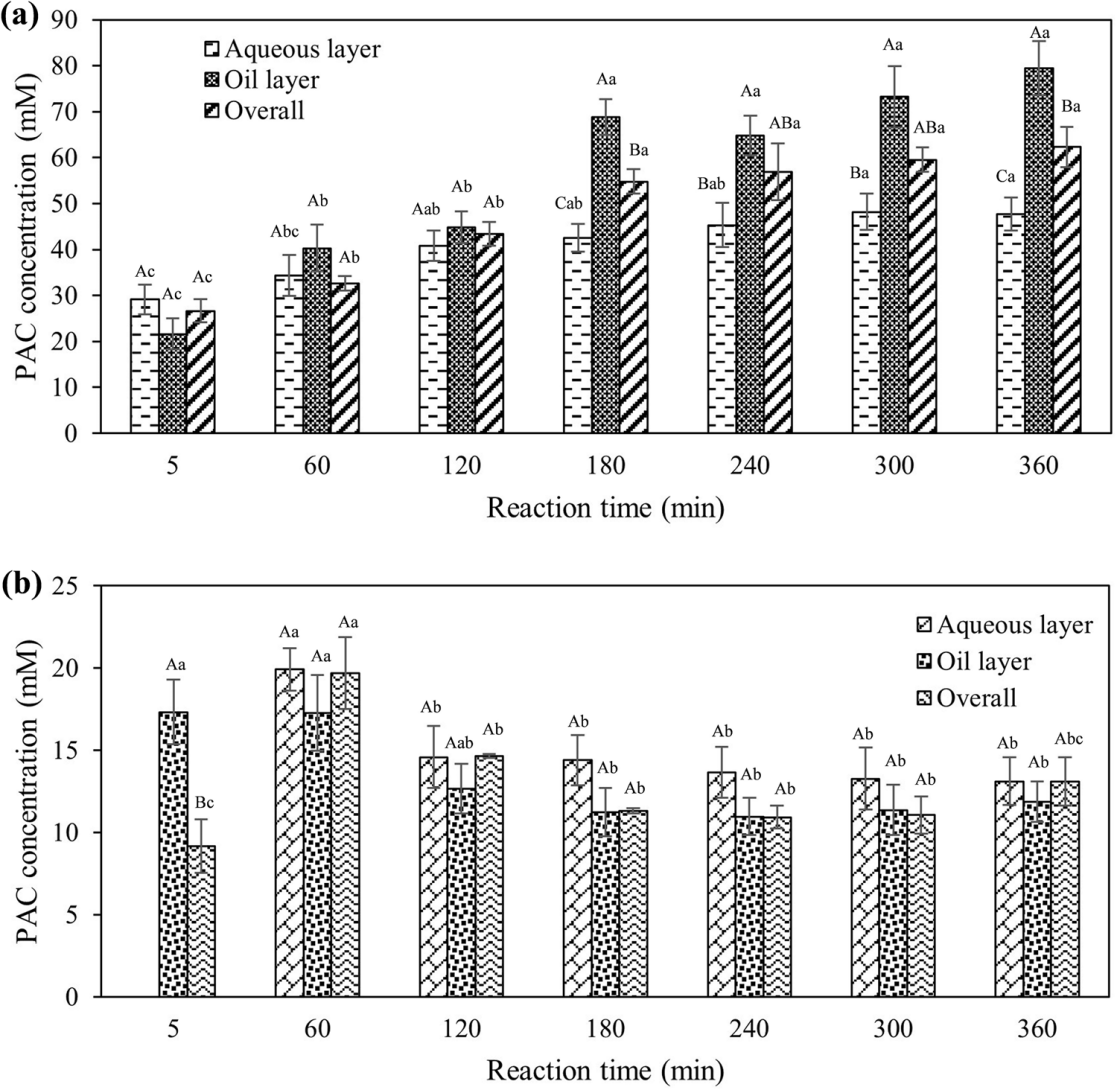
Biotransformation of phenylacetylcarbinol (PAC) in a two-layer system. (**a**) PAC concentration when whole cells biomass of *C. tropicalis* was used as biocatalyst; (**b**) PAC concentration when partially purified PDC was used as a biocatalyst. Error bars indicate the standard error from the mean (n = 3). Means with different uppercase alphabets indicated a significant difference (p ≤ 0.05) between aqueous, oil phase and overall, of the same reaction time. Means with different lowercase alphabets indicated a significant difference (p ≤ 0.05) between time intervals of the respective layer.

From the results, it was observed that the biomass of *C. tropicalis* (12.24 g L^−1^) equivalent to 1.29 ± 0.12 U mL^−1^ was able to produce PAC with an overall concentration of 32.6 ± 1.6 mM at 60 min and the concentration increased with the reaction time to reach twofold at the end of 6 h (Fig. 4a and Supplementary Table S6). In the other words, the whole cells *C. tropicalis* PDC at 1.29 U/mL produced a volumetric production of 1.56 g PAC L^−1^ h^−1^ at 10 °C with the specific productivity of 3.05 g g^−1^ biomass h^−1^. The organic phase accounted for significantly (p ≤ 0.05) higher PAC formation when compared to the aqueous phase as corroborating with the results of Rosche et al.^41^ who reported 687 mM PAC concentration in the organic octanol phase while the aqueous phase had only 86.7 mM. Similarly, Agustina et al.^50^ found that in the organic layer, the concentration of PAC was as high as 19.6 mM, while the buffer layer had only 1.76 mM.

In addition to the whole biomass of *C. tropicalis*, the PDC enzyme derived from its biomass was also utilized for the biotransformation process. The results showed that the overall highest PAC concentration was found to be 19.7 ± 2.2 mM at 60 min and decreased further as the reaction time increased to 6 h (Fig. 4b and Supplementary Table S7). It was observed that no significant (p > 0.05) difference in PAC concentration between the aqueous layer and organic layer of the biotransformation system. In contrast to the present study, Sandford et al.^84^ found that PAC production in a two-liquid system with partially purified PDC enzymes from *C. utilis* produced PAC up to 937 mM in the organic layer and 127 mM in the aqueous layer. It was also noted that in the present study, the biotransformation involved partially purified enzyme yielded less PAC when compared to the whole cells biomass of *C. tropicalis*. The higher PAC production achieved with whole cells PDC compared to partially purified PDC might be related to higher enzyme stability in the whole cells preparation^85^. This is due to the phospholipids as a cells component that acts as the barrier of the cells envelope and gives physical protection to enzymes inside cells^86^. Moreover, the cells-free enzyme preparation can result in PDC deactivation by the substrate benzaldehyde^87^. This is evident from a study conducted by Satianegara et al.^85^ who reported an 86% loss in the half-life of partially purified PDC compared to only 62% for whole cells preparation at 4 °C in the presence of 50 mM benzaldehyde.

In the present study, either whole cells biomass (12.24 g L^-1^) with initial volumetric PDC activity of 1.29 ± 0.12 U mL^−1^ or partially purified enzyme isolated from 12.24 g biomass with 0.32 ± 0.04 U mL^−1^ activity was used as biocatalysts. Although the initial PDC activity between the catalysts was unequal, it can be rationalized that enzyme purification process could incur higher cost and relatively high overall losses of enzyme activity to result in the equivalent quantity of enzyme. Thus, the intention of the study is to assess the catalysts for higher PAC production without incurring any additional costs. Moreover, utilization of whole cells has advantage over partially purified PDC as the former might offer higher catalyst stability than the enzymatic counterpart as partially purified PDC is more prone to deactivation effect by benzaldehyde^44^. In the case of both whole cells biomass and partially purified enzyme biocatalyst system, there was no formation of acetoin and benzyl alcohol as by-products when vegetable oil was used as an organic phase system. This is following Satianegara et al.^88^ who evaluated the pre-frozen whole cells biomass and partially purified enzyme of *C. utilis* and reported no formation of benzyl alcohol. However, in other studies which utilized immobilized whole cells^89^, the production of benzyl alcohol was witnessed. This might be due to the PAC formed within the first hour inhibited the formation of benzyl alcohol as it can inhibit the activity of alcohol dehydrogenase^90^ or there might be decrease in alcohol dehydrogenase activity due to the pre-frozen process^88^. From the comparison of whole cells and partially purified PDC in the PAC biotransformation, it is evident that utilization of whole cells as biocatalysts has considerable potential for cost reduction in term of appreciably higher PAC concentrations.

## Conclusion

The research explored the utilization of agro-based raw materials, SCB, SSB, CC and RS, for the production of ethanol and biotransformation of PAC. For this, different ethanol producing yeasts, *C. tropicalis*, *C. shehatae*, *S. cerevisiae* and *K. marxianus*, were evaluated for their potential. The study also compared the biotransformation process involving whole cells biomass with the process involving its partially purified enzyme for PAC production. In a nutshell, RS substrate fermented with *C. tropicalis* yielded higher ethanol (12.7 ± 0.23 g L^−1^) and specific PDC activity (0.351 ± 0.076 U mg^−1^ protein). Further, higher PAC formation (62.3 ± 4.4 mM) could be possible when the biotransformation process was carried out in a two-layer system with the whole cells biomass of *C. tropicalis* as a biocatalyst. Therefore, this study revealed that bioconversion of agro-industrial products would prove efficient in terms of alleviating waste disposal problems with the concomitant production of ethanol and phenylacetylcarbinol. The spent residue of the agro-materials after the enzymatic treatment remains to be evaluated for its compositional analysis and could be used as an animal feed supplement to generate zero waste from the process.

## Supplementary Information

Supplementary Tables.

## Data Availability

The data sets generated during and/or analysed during the current study are available from the corresponding authors on reasonable request.

## Abbreviations

PM: Particulate matter
PAC: Phenylacetylcarbinol
PDC: Pyruvate decarboxylase
YPD: Yeast extract-Peptone-Dextrose
Y_eth/s_: Yield of ethanol produced over total sugars consumed
Y_ace/s_: Yield of acetic acid produced over total sugars consumed
Y_x__/__s_: Yield of dried biomass produced over total sugars consumed
Q_p_: Volumetric productivity of ethanol per litre per hour; µ, specific growth rate (h^−^^1^)
q_s,Tot_: Specific total sugars consumption rate (g total sugars consumed g^−^^1^ biomass h^−^^1^)
HPLC: High Performance Liquid Chromatography
MOPS: 3-(*N*-Morpholino) propanesulfonic acid
