# Supplementary material for: Valorization of rice straw, sugarcane bagasse and sweet sorghum bagasse for the production of bioethanol and phenylacetylcarbinol
Source: Sci Rep. 2023 Jan 13;13:727. doi: 10.1038/s41598-023-27451-4 (PMC9839728; doi:10.1038/s41598-023-27451-4)
Supplement: Supplementary file 1 — Supplementary Tables. [file 41598_2023_27451_MOESM1_ESM.docx]

| **Substrate** | **Time (h)** | **pH** | **Total soluble solids (Brix^o^)** | **Dried biomass**  **(g L^-1^)** | **Concentration of sugars (g L^-1^)** | | | | | **Acetic acid (g L^-1^)** | **Ethanol**  **(g L^-1^)** |
| --- | --- | --- | --- | --- | --- | --- | --- | --- | --- | --- | --- |
|  |  |  |  |  | **Cellobiose** | **Glucose** | **Xylose** | **Arabinose** | **Total sugars** |  |  |
| Sugarcane bagasse (SCB) | 0 | **5.63^Aa^±0.04** | **11.1^Aa^±0.59** | 0.34^Ba^±0.02 | 0.00 | **47.3^Aa^±0.33** | 0.00 | 0.54^Bc^±0.01 | 47.9^Ab^±0.3 | 0.00 | 0.00 |
|  | 24 | 5.09^Bb^±0.003 | 7.23^Ba^±0.03 | 1.84^Ab^±0.09 | 0.00 | 2.95^Bc^±0.01 | 0.00 | 0.74^Ab^±0.01 | 3.69^Bb^±0.02 | 3.69^Bb^±0.08 | 17.7^Ab^±0.2 |
|  | 48 | 5.04^Ba^±0.05 | 6.67^Ba^±0.09 | 2.02^Ab^±0.06 | 0.00 | 2.56^Bb^±0.10 | 0.00 | 0.63^Bb^±0.04 | 3.19^Bb^±0.1 | **4.69^Aa^±0.27** | 16.6^Ba^±0.3 |
| Rice straw (RS) | 0 | **5.64^Aa^±0.003** | **11.7^Aa^±0.33** | 0.12^Cb^±0.03 | 0.62^Ab^±0.03 | **51.1^Aa^±2.56** | 10.2^Ab^±0.43 | 1.16^Aa^±0.04 | **63.1^Aa^±3.1** | 0.00 | 0.68^Ca^±0.10 |
|  | 24 | 5.15^Ba^±0.03 | 6.40^Bb^±0.15 | 2.19^Ba^±0.01 | 0.72^Aa^±0.11 | 7.38^Ba^±0.10 | 3.90^Ba^±1.97 | **1.48^Aa^±0.10** | 13.5^Ba^±1.9 | 5.13^Aa^±0.19 | **18.6^Aa^±0.2** |
|  | 48 | 5.05^Cb^±0.02 | 5.93^Bb^±0.03 | **2.46^Aa^±0.01** | 0.61^Aa^±0.09 | 7.15^Ba^±0.07 | 4.28^Ba^±1.51 | 1.29^Aa^±0.12 | 13.3^Ba^±1.6 | **5.60^Aa^±0.27** | 17.2^Ba^±0.2 |
| Sweet sorghum bagasse (SSB) | 0 | **5.67^Aa^±0.003** | **10.8^Aa^±0.73** | 0.26^Ca^±0.03 | **0.85^Aa^±0.02** | 31.3^Ab^±0.69 | **14.0^Aa^±0.29** | 0.86^Ab^±0.01 | 47.0^Ab^±1.0 | 0.00 | 0.75^Ca^±0.14 |
|  | 24 | 5.31^Ba^±0.04 | 6.40^Bb^±0.20 | 1.66^Bb^±0.12 | 0.46^Bb^±0.04 | 3.56^Bb^±0.05 | 3.06^Ba^±0.58 | 0.47^Bc^±0.05 | 7.55^Bb^±0.61 | 2.93^Bc^±0.18 | 12.9^Bc^±0.3 |
|  | 48 | 5.07^Ca^±0.02 | 5.17^Bc^±0.18 | 2.06^Ab^±0.06 | 0.20^Cb^±0.03 | 1.69^Cc^±0.04 | 0.09^Cb^±0.06 | 0.16^Cc^ ±0.02 | 2.15^Cb^±0.13 | **5.54^Aa^±0.34** | **14.7^Ab^±0.5** |

**Supplementary Table S1.** Changes in parameters during alcoholic fermentation of different substrates using *C. tropicalis.* Numbers with different uppercase alphabets in the same column indicated a significant difference (p ≤ 0.05) between time points of the same substrate. Numbers with different lowercase alphabets in the same column indicated a significant difference (p ≤ 0.05) between respective time points among substrates. The bolded and underlined numbers indicated the leading average value(s) among the samples of same time interval within the same column.

| **Substrate** | **Time (h)** | **Q_p_**  **(g_eth_ L^-1^ h^-1^)** | **µ**  **(h^-1^)** | **q_s,tot_**  **(g_s,tot_/g_x_.h)** | **Y_eth/s_**  **(g_eth/_g_s_)** | **Y_ace/s_**  **(g_ace/_g_s_)** | **Y_x/s_**  **(g_x/_g_s_)** |
| --- | --- | --- | --- | --- | --- | --- | --- |
| SCB | 0-24 | 0.738^Ab^±0.006 | 0.063^Ab^±0.004 | **1.24^Aa^±0.085** | **0.401^Aa^±0.001** | 0.084^Bb^±0.001 | 0.042^Aa^±0.002 |
|  | 0-48 | 0.346^Ba^±0.006 | 0.035^Bb^±0.001 | 0.555^Ba^±0.013 | 0.372^Aa^±0.003 | **0.105^Aa^±0.007** | **0.045^Aa^±0.001** |
| RS | 0-24 | **0.776^Aa^±0.010** | **0.086^Aa^±0.002** | **1.00^Aa^±0.11** | **0.382^Aa^±0.036** | 0.105^Aa^±0.010 | 0.045^Aa^±0.005 |
|  | 0-48 | 0.359^Ba^±0.005 | 0.049^Ba^±0.001 | 0.445^Bb^±0.028 | 0.348^Aa^±0.016 | **0.112^Aa^±0.002** | **0.050^Aa^±0.002** |
| SSB | 0-24 | 0.538^Ac^±0.013 | 0.058^Ab^±0.004 | **1.19^Aa^±0.10** | **0.329^Aa^±0.021** | 0.074^Bb^±0.002 | 0.042^Aa^±0.003 |
|  | 0-48 | 0.307^Bb^±0.010 | 0.038^Bb^±0.001 | 0.520^Ba^±0.019 | 0.329^Aa^±0.019 | **0.124^Aa^±0.010** | **0.046^Aa^±0.001** |

**Supplementary Table S2.** Comparison of kinetic parameters of batch cultivation processes during 0–48 h cultivation periods of *C. tropicalis* TISTR 5306 in different substrates. Numbers with different uppercase alphabets in the same column indicated a significant difference (p ≤ 0.05) between time intervals of the same substrate. Numbers with different lowercase alphabets in the same column indicated a significant difference (p ≤ 0.05) between respective time intervals among substrates. Y_eth/s_, mass yield of ethanol produced over total sugars consumed; Y_ace/s_, mass yield of acetic acid produced over total sugars consumed; Y_x/s_, mass yield of dried biomass produced over total sugars consumed; Q_p_, volumetric productivity of ethanol (g ethanol produced L^−1^ h^−1^); µ, specific growth rate (h^-1^); q_s,tot_, specific total sugars consumption rate (g total sugars consumed g^−1^ biomass h^−1^). The bolded and underlined numbers indicated the leading average value(s) among the samples of same time interval within the same column.

| **Yeast**  **species** | **Time (h)** | **pH** | **Total soluble solids**  **(Brix^o^)** | **Dried biomass**  **(g L^-1^)** | **Concentration of sugars (g L^-1^)** | | | | **Acetic acid (g L^-1^)** | **Ethanol**  **(g L^-1^)** |
| --- | --- | --- | --- | --- | --- | --- | --- | --- | --- | --- |
|  |  |  |  |  | **Cellobiose** | **Glucose** | **Xylose** | **Total sugars** |  |  |
| *C. tropicalis* | 0 | **5.87^Aa^±0.006** | **12.5^Aa^±0.29** | 0.11^Bb^±0.01 | **7.68^Aa^±0.15** | **39.8^Aa^±0.26** | **14.2^Aa^±0.46** | **61.6^Aa^±0.59** | 0.00 | 0.00 |
|  | 24 | 5.02^Bc^±0.003 | 9.20^Bd^±0.12 | **2.35^Aa^±0.09** | 7.13^Bb^±0.02 | 0.07^Bc^±0.01 | 13.8^Aa^±0.13 | 21.0^Bc^±0.14 | 3.26^Aa^±0.45 | **15.2^Aa^±0.34** |
|  | 48 | 5.01^Bc^±0.03 | 9.00^Bb^±0.01 | 2.38^Aa^±0.15 | 7.13^Bb^±0.02 | 0.03^Bbc^±0.01 | 9.87^Bc^±0.31 | 17.0^Cd^±0.29 | **5.39^Aa^±1.03** | 12.7^Ba^±0.23 |
| *C. shehatae* | 0 | 5.84^Ab^±0.003 | **12.7^Aa^±0.07** | 0.33^Ba^±0.14 | **7.45^Aa^±0.08** | **39.9^Aa^±1.09** | **15.0^Aa^±0.41** | **62.4^Aa^±1.35** | 0.00 | 0.00 |
|  | 24 | 5.15^Bb^±0.003 | 10.0^Bb^±0.03 | **2.50^Aa^±0.12** | 7.43^Aab^±0.16 | 24.1^Ba^±1.10 | 14.6^Aa^±0.14 | 46.2^Ba^±1.21 | 0.36^Bb^±0.03 | 5.42^Bc^±0.35 |
|  | 48 | 5.12^Bb^±0.03 | 9.33^Cab^±0.33 | 2.50^Aa^±0.08 | 7.42^Aa^±0.13 | 8.13^Ca^± 2.03 | 14.7^Aab^±0.05 | 30.8^Ca^±2.09 | 0.61^Ab^±0.05 | 11.1^Ac^±0.27 |
| *S. cerevisiae* | 0 | 5.83^Ab^±0.003 | **12.6^Aa^±0.01** | 0.04^Bb^±0.01 | **7.45^Aa^±0.08** | **40.3^Aa^±0.19** | **14.5^Aa^±0.29** | **62.2^Aa^±0.30** | 0.00 | 0.00 |
|  | 24 | 4.70^Bd^±0.007 | 9.47^Bc^±0.07 | **2.21^Aa^±0.43** | 7.50^Aa^±0.05 | 0.11^Bc^±0.01 | 14.2^Aa^±0.24 | 21.8^Bc^±0.24 | 3.35^Ba^±0.68 | **14.5^Aa^±0.08** |
|  | 48 | 4.41^Cd^±0.007 | 9.40^Bab^±0.12 | 2.37^Aa^±0.19 | 7.50^Aa^±0.02 | 0.08^Bb^±0.01 | 14.2^Ab^±0.25 | 21.8^Bc^±0.23 | **5.36^Aa^±0.74** | 9.22^Bd^±0.13 |
| *K. marxianus* | 0 | 5.82^Ac^± < 0.001 | **12.6^Aa^±0.23** | 0.05^Bb^±0.01 | **7.54^Aa^±0.05** | **39.2^Aa^±0.37** | **14.8^Aa^±0.71** | **61.6^Aa^±0.66** | 0.00 | 0.00 |
|  | 24 | 5.28^Ba^±0.003 | 11.2^ABa^±0.01 | 1.15^Ab^±0.08 | 7.54^Aa^±0.07 | 15.3^Bb^±0.18 | 14.9^Aa^±0.70 | 37.8^Bb^±0.66 | 1.39^Bb^±0.73 | 6.93^Bb^±0.05 |
|  | 48 | 5.26^Ca^± < 0.001 | 9.67^Ca^±0.07 | 1.29^Ab^±0.02 | 7.49^Aa^±0.03 | 0.01^Cc^±0.01 | 15.1^Aa^±0.24 | 22.6^Cb^±0.22 | **3.57^Aa^±0.22** | 11.8^Ab^±0.17 |

**Supplementary Table S3.** Changes in parameters during alcoholic fermentation by different yeasts using rice straw as a substrate. Numbers with different uppercase alphabets in the same column indicated a significant difference (p ≤ 0.05) between time points of the same yeast species. Numbers with different lowercase alphabets in the same column indicated a significant difference (p ≤ 0.05) between respective time points among yeast species. The bolded and underlined numbers indicated the leading average value(s) among the samples of same time interval within the same column.

| **Substrate** | **Time (h)** | **Q_p_**  **(g_eth_ L^-1^ h^-1^)** | **µ**  **(h^-1^)** | **q_s,tot_**  **(g_s,tot_/g_x_.h)** | **Y_eth/s_**  **(g_eth/_g_s_)** | **Y_ace/s_**  **(g_ace/_g_s_)** | **Y_x/s_**  **(g_x/_g_s_)** |
| --- | --- | --- | --- | --- | --- | --- | --- |
| *C. tropicalis* | 0-24 | **0.635^Aa^±0.014** | **0.094^Aa^±0.004** | **0.758^Aa^±0.045** | **0.375^Aa^±0.006** | 0.081^Aa^±0.013 | 0.058^Ab^±0.003 |
|  | 0-48 | 0.264^Ba^±0.005 | 0.047^Ba^±0.003 | 0.411^Bb^±0.018 | 0.284^Bb^±0.005 | **0.122^Aa^±0.025** | 0.053^Ab^±0.002 |
| *C. shehatae* | 0-24 | 0.226^Ac^±0.014 | **0.090^Aa^±0.006** | 0.373^Ab^±0.060 | **0.352^Aa^±0.063** | 0.023^Ab^±0.005 | **0.161^Aa^±0.025** |
|  | 0-48 | 0.231^Ac^±0.006 | 0.045^Ba^±0.001 | 0.378^Ab^±0.050 | 0.346^Aa^±0.023 | 0.019^Ab^±0.002 | 0.078^Ba^±0.005 |
| *S. cerevisiae* | 0-24 | **0.606^Aa^±0.003** | **0.090^Aa^±0.017** | **0.839^Aa^±0.160** | **0.360^Aa^±0.001** | 0.083^Ba^±0.017 | 0.055^Ab^±0.011 |
|  | 0-48 | 0.192^Bd^±0.003 | 0.048^Ba^±0.004 | 0.367^Bb^±0.029 | 0.228^Bc^±0.005 | **0.132^Aa^±0.017** | 0.058^Ab^±0.004 |
| *K. marxianus* | 0-24 | 0.289^Ab^±0.002 | 0.046^Ab^±0.003 | **0.911^Aa^±0.083** | **0.292^Aa^±0.015** | 0.062^Ba^±0.035 | 0.048^Ab^±0.005 |
|  | 0-48 | 0.246^Bb^±0.004 | 0.026^Bb^±0.000 | 0.655^Ba^±0.010 | 0.303^Ab^±0.003 | **0.092^Aa^±0.005** | 0.033^Bc^±0.001 |

**Supplementary Table S4.** Comparison of kinetic parameters of batch cultivation processes during 0–48 h cultivation periods of different yeasts with rice straw as substrate. Numbers with different uppercase alphabets in the same column indicated a significant difference (p ≤ 0.05) between time intervals of the same yeast. Numbers with different lowercase alphabets in the same column indicated a significant difference (p ≤ 0.05) between respective time intervals among yeasts. Y_eth/s_, yield of ethanol produced over total sugars consumed; Y_ace/s_, yield of acetic acid produced over total sugars consumed; Y_x/s_, yield of dried biomass produced over total sugars consumed; Q_p_, volumetric productivity of ethanol per litre per hour; µ, specific growth rate (h^-1^); q_s,tot_, specific total sugars consumption rate (g total sugars consumed g^−1^ biomass h^−1^). The bolded and underlined numbers indicated the leading average value(s) among the samples of same time interval within the same column.

| **Yeast**  **species** | **Time (h)** | **Volumetric PDC enzyme activity (U mL^-1^)** | **Specific PDC enzyme activity (U mg^-1^)** |
| --- | --- | --- | --- |
|  |  |  |  |
| *C. tropicalis* | 0 | 0.029^Cc^±0.002 | 0.167^Bb^±0.008 |
|  | 24 | **0.303^Aa^±0.020** | **0.469^Aa^±0.031** |
|  | 48 | 0.257^Ba^±0.008 | 0.415^Aa^±0.041 |
| *C. shehatae* | 0 | 0.060^Aa^±0.004 | 0.386^Aa^±0.025 |
|  | 24 | 0.053^ABc^±0.004 | 0.295^Bb^±0.005 |
|  | 48 | 0.045^Bd^±0.003 | 0.252^Bb^±0.026 |
| *S. cerevisiae* | 0 | 0.050^Cab^±0.003 | 0.194^Ab^±0.028 |
|  | 24 | 0.212^Ab^±0.011 | 0.146^ABc^±0.004 |
|  | 48 | 0.115^Bc^±0.009 | 0.121^Bc^±0.012 |
| *K. marxianus* | 0 | 0.042^Bb^±0.004 | 0.176^ABb^±0.010 |
|  | 24 | 0.208^Ab^±0.012 | 0.271^Ab^±0.055 |
|  | 48 | 0.203^Ab^±0.024 | 0.117^Bc^±0.008 |

**Supplementary Table S5.** Intracellular pyruvate decarboxylase (PDC) activity of yeasts during cultivation with rice straw as substrate. Numbers with different uppercase alphabets in the same column indicated a significant difference (p ≤ 0.05) between time points of the same yeast species. Numbers with different lowercase alphabets in the same column indicated a significant difference (p ≤ 0.05) between respective time points among yeast species. The bolded and underlined numbers indicated the leading average value(s) among the samples of same time interval within the same column.

| **Time (min)** | **PAC concentration (mM)** | | |
| --- | --- | --- | --- |
|  | **Aqueous layer** | **Oil layer** | **Overall** |
| 0 | 0.00±0.00 | 0.00±0.00 | 0.00±0.00 |
| 5 | 25.1^Ac^±4.73 | 21.6^Ac^±3.38 | 26.6^Ac^±2.53 |
| 60 | 30.2^Abc^±5.37 | 40.3^Ab^±5.07 | 32.6^Ab^±1.55 |
| 120 | **40.8^Aab^±3.31** | 44.8^Ab^±3.42 | 43.4^Ab^±2.59 |
| 180 | **42.6^Cab^±2.97** | **68.8^Aa^±3.87** | **54.8^Ba^±2.68** |
| 240 | **45.3^Bab^±4.77** | **64.8^Aa^±4.27** | **56.9^ABa^±6.18** |
| 300 | **48.2^Ba^±3.97** | **73.2^Aa^±6.62** | **59.5^ABa^±2.66** |
| 360 | **47.7^Ca^±3.60** | **79.4^Aa^±5.93** | **62.3^Ba^±4.37** |

**Supplementary Table S6.** Biotransformation of phenylacetylcarbinol (PAC) in a two-layer system using whole cell biomass of *C. tropicalis* as biocatalyst. Numbers with different uppercase alphabets in the same column indicated a significant difference (p ≤ 0.05) between time points. Numbers with different lowercase alphabets in the same row indicated a significant difference (p ≤ 0.05) between aqueous, oil phase and overall, at respective time points. The bolded and underlined numbers indicated the leading average value(s) within the same column.

| **Time (min)** | **PAC concentration (mM)** | | |
| --- | --- | --- | --- |
|  | **Aqueous layer** | **Oil layer** | **Overall** |
| 0 | 0.00±0.00 | 0.00±0.00 | 0.00±0.00 |
| 5 | 0.00±0.00 | **17.3^Aa^±1.96** | 9.17^Bc^±1.64 |
| 60 | **19.9^Aa^±1.28** | **17.3^Aa^±2.30** | **19.7^Aa^±2.18** |
| 120 | 14.6^Ab^±1.90 | **12.7^Aab^±1.52** | 14.6^Ab^±0.11 |
| 180 | 14.4^Ab^±1.53 | 13.2^Ab^±2.26 | 11.3^Ab^±0.16 |
| 240 | 13.7^Ab^±1.55 | 12.2^Ab^±2.03 | 10.9^Ab^±0.69 |
| 300 | 13.3^Ab^±1.87 | 12.9^Ab^±2.97 | 11.1^Ab^±1.12 |
| 360 | 13.1^Ab^±1.46 | 11.9^Ab^±1.22 | 13.1^Abc^±1.48 |

**Supplementary Table S7.** Biotransformation of phenylacetylcarbinol (PAC) in a two-layer system using partially purified PDC as biocatalyst. Numbers with different uppercase alphabets in the same column indicated a significant difference (p ≤ 0.05) between time points. Numbers with different lowercase alphabets in the same row indicated a significant difference (p ≤ 0.05) between aqueous, oil phase and overall, at respective time points. The bolded and underlined numbers indicated the leading average value(s) within the same column.
